# A New Player in Jasmonate-Mediated Stomatal Closure: The *Arabidopsis thaliana* Copper Amine Oxidase β

**DOI:** 10.3390/cells10123399

**Published:** 2021-12-02

**Authors:** Ilaria Fraudentali, Chiara Pedalino, Paraskevi Tavladoraki, Riccardo Angelini, Alessandra Cona

**Affiliations:** 1 Department of Science, University “Roma Tre”, 00146 Rome, Italy; ilaria.fraudentali@uniroma3.it (I.F.); chi.pedalino@stud.uniroma3.it (C.P.); paraskevi.tavladoraki@uniroma3.it (P.T.); riccardo.angelini@uniroma3.it (R.A.); 2 Interuniversity Consortium National Institute of Biostructures and Biosystems (INBB), 00136 Rome, Italy

**Keywords:** copper amine oxidases, polyamines, hydrogen peroxide, hormones, methyl jasmonate, stomatal closure, stress response

## Abstract

Plant defence responses to adverse environmental conditions include different stress signalling, allowing plant acclimation and survival. Among these responses one of the most common, immediate, and effective is the modulation of the stomatal aperture, which integrates different transduction pathways involving hydrogen peroxide (H_2_O_2_), calcium (Ca^2+^), nitric oxide (NO), phytohormones and other signalling components. The *Arabidopsis thaliana copper amine oxidases β* (*AtCuAOβ*) encodes an apoplastic CuAO expressed in guard cells and root protoxylem tissues which oxidizes polyamines to aminoaldehydes with the production of H_2_O_2_ and ammonia. Here, its role in stomatal closure, signalled by the wound-associated phytohormone methyl-jasmonate (MeJA) was explored by pharmacological and genetic approaches. Obtained data show that *AtCuAOβ* tissue-specific expression is induced by MeJA, especially in stomata guard cells. Interestingly, two *Atcuaoβ* T-DNA insertional mutants are unresponsive to this hormone, showing a compromised MeJA-mediated stomatal closure compared to the wild-type (WT) plants. Coherently, *Atcuaoβ* mutants also show compromised H_2_O_2_-production in guard cells upon MeJA treatment. Furthermore, the H_2_O_2_ scavenger *N,N^1^*-dimethylthiourea (DMTU) and the CuAO-specific inhibitor 2-bromoethylamine (2-BrEtA) both reversed the MeJA-induced stomatal closure and the H_2_O_2_ production in WT plants. Our data suggest that *AtCuAOβ* is involved in the H_2_O_2_ production implicated in MeJA-induced stomatal closure.

## 1. Introduction

Growing anthropogenic pressure, ongoing climate changes and the intensification of extreme events expose plant organisms to unusual and unpredictable environmental conditions, subjecting them to intense abiotic stresses, greatly varying in intensity, frequency and duration. As a result of non-optimal growth conditions, plants develop an increased vulnerability to pathogens and pests. Stomata play a pivotal role in the interaction between plants and the environment and are responsible for the balance between water loss and gas exchange. The regulation of stomatal movement represents the most immediate and effective strategy to promptly respond to climatic changes. In the current context of dramatic environmental changes, such as the increase in atmospheric CO_2_, which is strongly responsible for the rise in temperature and the decrease in water resources, the understanding of the modulation of stomata responses is of great importance in the design of sustainable agriculture, which requires new varieties with improved growth-water loss trade-off. Stomatal movement is a complex physiological event evolved to regulate gas exchanges and thermoregulation, finely modulated by different exogenous factors, such as light, temperature, drought, and pathogens. In this context, mechanical stresses caused by atmospheric agents or biotic factors, such as herbivorous animals, leaf-chewing or sucking insects and root nematodes, lead to tissue damage, requiring an immediate array of molecular responses to limit the injury damage and/or leading to increased defence capacity towards pests.

Wounding response involves the phytohormone jasmonate (JA) and its derivatives and induces the production of hydrogen peroxide (H_2_O_2_) in guard cells, which in turn induces an increase in intracellular nitric oxide (NO) and calcium (Ca^2+^) levels, leading to stomatal closure [1]. A well-known source of reactive oxygen species (ROS) in the cell-wall of guard cells is represented by plasma membrane NADPH oxidases, but recently it has been shown that copper amine oxidases (CuAOs) and FAD-dependent polyamine oxidases (PAOs) involved in polyamine (PA) oxidation may also contribute to H_2_O_2_ production [2,3,4].

Supporting this hypothesis, it has been shown that in fava bean (*Vicia faba*) abscisic acid (ABA)-induced stomatal closure implies CuAO-mediated H_2_O_2_ production in the apoplast, which contributes to an increase in the cytosolic Ca^2+^ levels in response to ABA [5]. In addition, it has been described that PAOs contribute to the control of stomatal movement in grapevine (*Vitis vinifera*) and Arabidopsis [6,7]. Coherently, evidence of the AOs involvement in the regulation of stomatal movement has been reported. The peroxisomal AtCuAOζ and the vacuolar AtCuAOδ were shown to be involved in the ABA-mediated control of stomatal closure [3,4]. The constitutive expression of *AtCuAOβ* in leaf and flower guard cells, together with its induction in the same organs upon treatment with the stress-related hormone methyl jasmonate (MeJA) [8,9,10], suggesting that AtCuAOβ has a role in the regulation of stomatal aperture levels under MeJA-signalled stress conditions, leading us to study the involvement of this protein as a H_2_O_2_ source in the MeJA-induced stomatal closure.

## 2. Materials and Methods

### 2.1. Plant Materials, Growth Conditions and Treatments

The Columbia-0 (Col-0) ecotype of *Arabidopsis thaliana* was used as the wild type (WT). The Arabidopsis Col-0 T-DNA insertion lines *Atcuaoβ.1* (SALK_145639.55.25.x; TAIR accession number 1005841762) and *Atcuaoβ.3* (SALK_082394.32.30.x, TAIR accession number 1005822711) of the *CuAO* gene At4g14940 (*AtCuAOβ*, TAIR accession no. 2129519) used were obtained from the SALK Institute Genomic Analysis Laboratory (http://signal.salk.edu/tabout.html accessed on 15 September 2021; Alonso et al., 2003) and characterized (*Atcuaoβ.1* [9]; *Atcuaoβ.3*: Figure S1). Transgenic plants *AtCuAOβ-promoter::GFP-GUS* analysed were previously described [8,9].

Plants were grown in a growth chamber at 23 °C under long-day conditions (16/8 h photoperiod; 50 μmol m^−2^ s^−1^ and 55% relative humidity). For in vitro growth, seeds were surface sterilized as previously described [4,9,11,12]. Seeds were stratified at 4 °C for 2 days in the dark and then sown in ½ Murashige and Skoog (MS) salt mixture (pH 5.7) supplemented with 0.5 (*w*/*v*) sucrose, 0.8% (*w*/*v*) agar (solid medium) and 50 µg/mL kanamycin (when antibiotic selection was necessary).

*AtCuAOβ* gene RT-quantitative PCR (RT-qPCR) analysis of MeJA treatments were performed on seven-day-old WT seedlings grown for six days in solid medium and then transferred to ½ MS salt mixture (pH 5.7) supplemented with 0.5 (*w*/*v*) sucrose (liquid medium) for one more day, as acclimation. After this period, liquid medium was replaced by fresh liquid medium containing 50 μM MeJA (Duchefa, Haarlem, The Netherlands), using fresh liquid medium alone for control. Plant samples for gene expression studies were harvested at the described times, frozen in liquid nitrogen and then kept at −80 °C until RNA extraction.

The histochemical GUS analysis was performed on seven-day-old seedlings grown on solid medium supplemented with kanamycin as hereafter described. In detail, for the time course analysis of inducible tissue-specific gene expression after MeJA treatment *AtCuAOβ-promoter::GFP-GUS* six-day-old seedlings were transferred to 12-well tissue culture clusters containing liquid medium for one day. Successively fresh liquid medium was supplemented or not with MeJA 50 μM and treatment was allowed to proceed for 5 min, 15 min, 30 min, 1 h, 3 h, 6 h and 24 h (time course analysis). Samples were analysed under light microscope (LM).

Stomatal aperture measurements were performed on seven-day-old WT plants and *Atcuaoβ* mutants, grown on solid medium under control conditions or after treatment with MeJA (0.5, 5 and 50 μM), *N,N^1^*-dimethylthiourea (DMTU; 100 μM), H_2_O_2_ (1, 10 and 100 μM), and 2-bromoethylamine (2-BrEtA; 5 mM), conducted alone or in combination, as described further below.

The detection of ROS in guard cells was analysed on seven-day-old WT and *Atcuaoβ* mutant seedlings grown on solid medium and examined under control conditions and after treatment with 50 μM MeJA, 50 μM MeJA + 100 μM DMTU, 50 μM MeJA + 5 mM 2-BrEtA.

### 2.2. Identification of the T-DNA Insertional Loss-of-Function Atcuaoβ.3 Mutant

Plants homozygous for the T-DNA insertion were identified by PCR on total DNA extracted from leaves by alkali treatment [13] using gene- and T-DNA-specific primers (Table 1). *Atcuaoβ.3* gene-specific primers (*RP-Atcuaoβ.3*/*LP-Atcuaoβ.3*) were designed outside of the T-DNA insertion, and the T-DNA-specific primer (*LBa1*) was designed at its left border (Figure S1). The genotype of the *Atcuaoβ.3* mutants was ascertained by two sets of PCRs: one using *RP-Atcuaoβ.3*/*LBa1* that determines the presence of the T-DNA insertion, and the other using *RP-Atcuaoβ.3*/*LP-Atcuaoβ.3* that verifies the absence of the fragment indicative of a WT allele, as the T-DNA insertion originates a non-amplifiable long transcript. Moreover, quantitative expression profiles of *AtCuAOβ* were determined by RT-qPCR on seven-day-old WT and *Atcuaoβ.3* whole seedlings to verify the absence of *AtCuAOβ* gene expression (Figure S1), as described further below.

### 2.3. RNA Extraction, RT-PCR and RT-Quantitative PCR (RT-qPCR)

Total RNA was isolated from WT seedlings (100 mg) by using TRIzol^®^ Reagent (Invitrogen, Carlsbad, CA, USA) following the manufacture’s instruction with slight modifications, as described elsewhere [4,12].

Quantitative expression profiles of *AtCuAOβ* were determined by RT-qPCR on seven-day-old whole seedlings after treatment with MeJA 50 μM using a Corbett Rotor-Gene 6000 (Corbett Life Science, QIAGEN, Venlo, The Netherlands), as described elsewhere [4,12]. In detail, RT-qPCR analysis was performed on DNase-treated RNA (4 μg) as follows. cDNA synthesis and PCR amplification were carried out using *GoTaq^®^ 2-Step RT-qPCR System200* (Promega, Madison, WI, USA) following manufacturer’s protocol. The first cDNA strand was synthesized using random and oligo *dT* primers in an *iCycler TM Thermal Cycler* (Bio-Rad, Hercules, CA, USA) with the following parameters: 25 °C for 5 min, 42 °C for 60 min and 70 °C for 15 min. The PCRs were run in a Corbett RG6000 (Corbett Life Science, QIAGEN) utilizing the following program: 95 °C for 2 min then 40 cycles of 95 °C for 7 s and 60 °C for 40 s. The melting program ramps from 60 °C to 95 °C rising by 1 °C each step. *AtCuAOβ* specific primers were *AtCuAOβ-qPCR-for*/*rev* (Table 2). *Ubiquitin-conjugating enzyme 21* (*UBC21*, At5g25760) was used as reference gene and specific primers were prepared [14] (*UBC21-for* and *UBC21-rev*; Table 2). The software controlling the thermocycler and data analysis was the Corbett Rotor-Gene 6000 Application Software (version 1.7, Build 87; Corbett Life Science, QIAGEN). Fold change in the expression of the *AtCuAOβ* gene was calculated according to the ΔΔCq method as previously described [4,15].

### 2.4. Histochemical Analysis of GUS Assay

GUS staining was performed as previously described [16] with modifications [9,12]. In detail, for time course analysis, after 50 μM MeJA treatment, samples were gently soaked in ice cold 90% (*v*/*v*) acetone for 30 min for prefixation, rinsed three times with sodium phosphate buffer (50 mM, pH 7.0) and then immersed in staining solution [1 mM 5-bromo-4-chloro-3-indolyl-β-d-glucuronide, 2.5 mM potassium ferrocyanide, 2.5 mM potassium ferricyanide, 0.1% (*v*/*v*) Triton X-100, 10 mM EDTA in sodium phosphate buffer (50 mM, pH 7.0)]. Histochemical GUS staining was allowed to proceed until differences in the intensity between treated and untreated plants were detected under the microscope (2 h). For developmental tissue-specific gene expression, the reaction proceeded overnight at 37 °C in dark. Chlorophyll was extracted by washing in sequence with ethanol/acetic acid ratio 1:3 (*v*/*v*) for 30 min, ethanol/acetic acid ratio 1:1 (*v*/*v*) for 30 min and with 70% ethanol for another 30 min. Samples were stored in 70% ethanol at 4 °C, prior to being observed under LM. Images were acquired by a Leica DFC450C digital camera applied to a Zeiss Axiophot 2 microscope.

### 2.5. Measurement of Stomatal Aperture

Measurement of stomatal aperture was performed as previously described [17], with modifications [4] (Figure S2). In detail, seven-day-old WT and *Atcuaoβ* mutant seedlings grown on solid medium were incubated in opening solution (30 mM KCl, 10 mM MES-Tris, pH 6.15) for 3 h under light to allow stomatal opening. After this period, the opening solution was replaced by liquid medium (protocol modification described in Supplementary Materials section, Figure S2) in the absence or presence of treatment performed as follows: MeJA 0.5, 5 or 50 μM; DMTU 100 μM; MeJA 0.5, 5 or 50 μM + DMTU 100 μM; MeJA 50 μM + 2-BrEtA 5 mM, H_2_O_2_ 1, 10 or 100 μM. Seedlings were incubated for 1 h (dose–response curve analysis) or 15 min, 30 min, 1 h, 3 h and 24 h (time course analysis) under light. Following the various treatments, seedlings were incubated for 30 min under light in a fixing solution (1% glutaraldehyde, 10 mM NaPi pH 7.0, 5 mM MgCl_2_, and 5 mM EDTA). Stomata images with the outline of the pores in the focal plane were acquired by a Leica DFC 450C digital camera applied to a Zeiss Axiophot 2 microscope at the magnification of 20×. Stomata pores width and length were measured using a digital ruler (ImageJ 1.44) and their apertures were expressed as a width/length ratio.

### 2.6. In Situ Detection of Reactive Oxygen Species (ROS) in Guard Cells

ROS production in guard cells was analysed using a chloromethyl derivative of 2′,7′-dichlorodihydrofluorescein diacetate (CM-H_2_DCFDA; Molecular Probes, Invitrogen) as previously described [4] with slight modifications. Seven-day-old WT and *Atcuaoβ* mutant seedlings were incubated for 3 h in the assay solution containing 5 mM KCl, 50 μM CaCl_2_ and 10 mM MES-Tris (pH 6.15), and then 10 μM CM-H_2_DCFDA was added to the sample. Seedlings were incubated for 30 min at room temperature and then the excess dye was washed out twice with the assay solution. After this period, the assay solution was replaced by liquid medium in the absence or presence of treatment performed as follows: 50 μM MeJA, 50 μM MeJA + 100 μM DMTU, 50 μM MeJA + 5 mM 2-BrEtA. Seedlings were incubated for 1 h. Images were acquired by Laser Scanning Confocal Microscopy (LSCM), using a Leica TCS-SP5 equipped with an Argon laser (Excitation/Emission: ~492–495/517–527 nm) and the Leica Application Suite Advanced Fluorescence (LAS-AF; Leica Microsystems, Wetzlar, Germany).

### 2.7. Statistics

The RT-qPCR analysis was performed on three biological replicates, each with three technical replicates (*n* = 3). The analysis by GUS staining of tissue-specific gene expression was performed on a minimum of fifteen plants from three independent experiments. Images from a single representative experiment are shown. For the stomatal aperture measurements, three independent experiments were performed for each treatment on the different genotypes. For each time-point, five similarly sized leaves were harvested from different seedlings for each genotype and treatment. In this case, each of the five leaves from the three experiments was considered a biological replicate for a total of fifteen biological replicates for each genotype and treatment (*n* = 15). For each leaf, four random chosen fields (430 μm × 325 μm) were acquired and at least 60 stomata were measured. The mean values were used in the statistical analysis. LSCM analysis of the CM-H_2_DCFDA staining was performed on seedlings from five independent experiments, each time analysing five similarly sized leaves harvested from different seedlings for each genotype and treatment. Images from a single representative experiment are shown.

Statistical tests of RT-qPCR and stomatal aperture were performed using GraphPad Prism (GraphPad Software) with One-way ANOVA analysis followed by Sidak’s multiple comparison tests. Statistical significance of differences was evaluated by *p* levels. *ns*, not significant *p* levels > 0.05; *, **, *** and **** *p* levels ≤ 0.05, 0.01, 0.001 and 0.0001, respectively.

## 3. Results

### 3.1. Tissue-Specific Expression Profile of AtCuAOβ in Stomata Guard Cells

**Figure 1:**
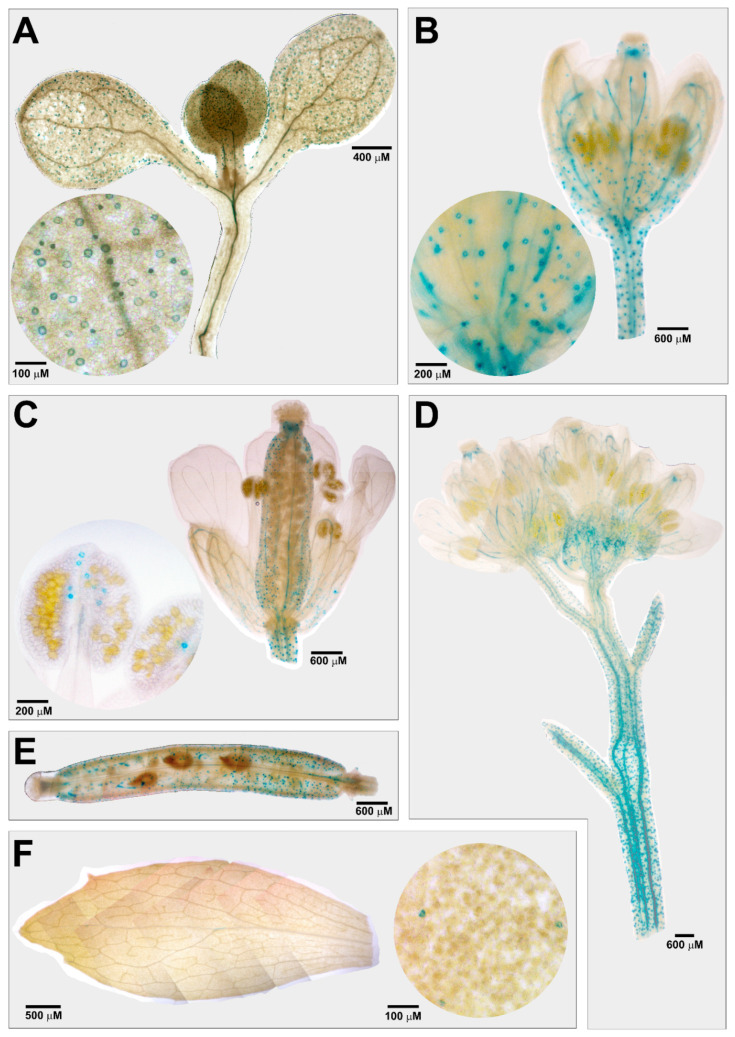
*AtCuAOβ* tissue expression pattern in stomata of seven-day-old seedling (**A**) and in flowers (**B**–**D**), siliques (**E**) and leaves (**F**) of *AtCuAOβ::GFP-GUS* adult transgenic plants. The GUS staining reaction was allowed to proceed overnight. Highly reproducible results were obtained from three independent transgenic lines. Shown images were obtained aligning serial overlapping micrographs of the same samples by Photoshop Software (Adobe) and are representative of those obtained from 25 samples from five independent experiments. The circular images show the enlarged detail (stomata guard cells) of the corresponding representations in the inset.

As previously described, *AtCuAOβ* encodes an apoplastic CuAO expressed at the early stages of vascular tissue differentiation in root, as well as in stomata guard cells [8,9]. Here, to integrate information from previous reported data, *AtCuAOβ* expression pattern has been analysed in seedlings and in leaves, flowers and siliques of *AtCuAOβ-promoter::GFP-GUS* Arabidopsis adult transgenic plants. Analysis of promoter-driven GUS staining revealed a strong *AtCuAOβ* expression in guard cells of different tissues/organs in different developmental stages, such as cotyledonary leaves from seven-day-old seedlings and leaves, pedicels, sepals, styles, valves and anther epidermis from adult transgenic plants (Figure 1). Moreover, *AtCuAOβ* expression is also present in vascular tissues of hypocotyl, young leaves, sepals, pedicels, stem, and replum (Figure 1).

### 3.2. Expression Profile of AtCuAOβ after MeJA Treatment

**Figure 2:**
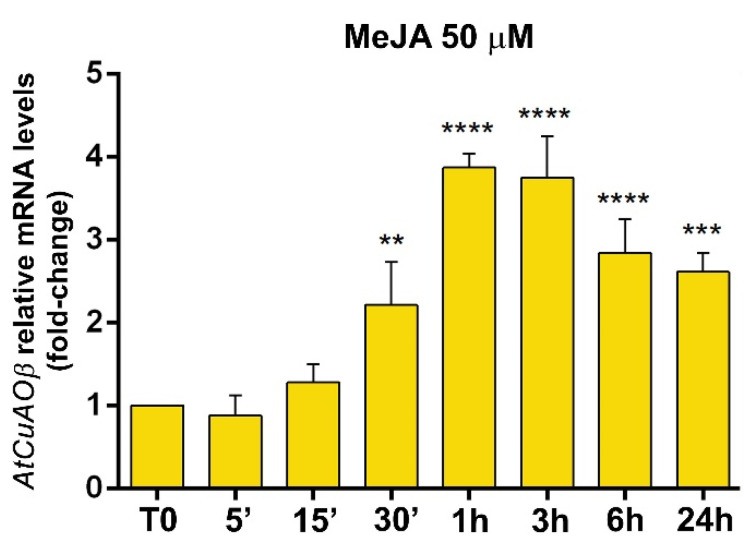
Time course analysis of *AtCuAOβ* gene expression by RT-qPCR after 50 μM MeJA treatment. Gene expression was analysed in seven-day-old WT seedlings untreated or treated for 0 h (T0), 5, 15, 30 min 1, 3, 6 and 24 h. The reported values of expression fold-inductions after treatment are relative to the corresponding expression values of non-treated plants for each time point, with the value for time zero assumed to be one. Data are the result of three biological replicates, each with three technical replicates (mean values ± SD; *n* = 3). The significance levels between relative mRNA levels at each time point and time 0 are reported only when *p* ≤ 0.05. **, ***, **** *p* levels ≤ 0.01, 0.001 and 0.0001, respectively.

*CuAO* expression has been reported to be induced by exogenously supplied JA and MeJA in several plant species [12,18,19]. In order to integrate information from previous reported data [9], a 24 h time course analysis of the modulation of *AtCuAOβ* expression by 50 µM MeJA treatment was carried out by RT-qPCR analysis (Figure 2). *AtCuAOβ* expression was induced by MeJA treatment of 2-fold after 30 min, strongly peaked at 4-fold from 1 to 3 h, and then progressively decreased towards 24 h, where a 2.7-fold induction was still observed, compared to T0 levels (Figure 2).

**Figure 3:**
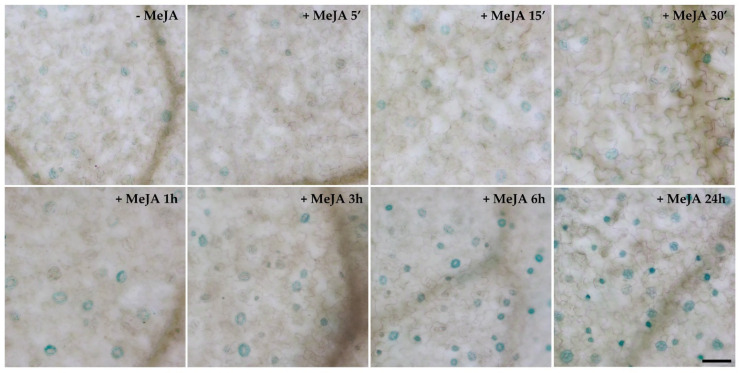
Analysis of *AtCuAOβ* tissue-specific expression pattern upon treatment with 50 μM MeJA. Light microscopy analysis by GUS staining in cotyledons of seven-day-old *AtCuAOβ*::*GFP-GUS* transgenic seedlings untreated (Figure S3) or treated with 50 μM MeJA for 5 min, 15 min, 30 min, 1 h, 3 h, 6 h and 24 h. The staining reaction proceeded for 2 h. Micrographs are representative of those obtained from fifteen leaves from three independent experiments. Bar = 50 µm.

It was previously demonstrated that *AtCuAOβ* expression is strongly induced by the wound-signal hormone MeJA in the root vascular tissues [9]. Herein, the time course analysis of tissue-specific expression pattern of *AtCuAOβ* after MeJA treatment was extended to other organs in *AtCuAOβ-promoter::GFP-GUS* plants. MeJA induced *AtCuAOβ* expression also in stomata of cotyledons, as revealed by the presence of a time-dependent increase in GUS staining intensity in stomata guard cells in treated-seedlings (Figure 3) as compared to control plants (Figure 3 and Figure S3).

### 3.3. AtCuAOβ-Dependent H_2_O_2_ Production Is Involved in MeJA-Induced Stomatal Closure

The constitutive expression of *AtCuAOβ* in guard cells of leaves and flowers (Figure 1), together with its induction in the same organs after treatment with the stress-related hormone MeJA would suggest a role of *AtCuAOβ* in the regulation of stomatal aperture levels under stress conditions. Considering this hypothesis, studies on the involvement of *AtCuAOβ* in the regulation of stomatal movement under MeJA treatment was performed, using WT plants and two different *Atcuaoβ* loss-of-function mutants, *Atcuaoβ.1* and *Atcuaoβ.3*.

**Figure 4:**
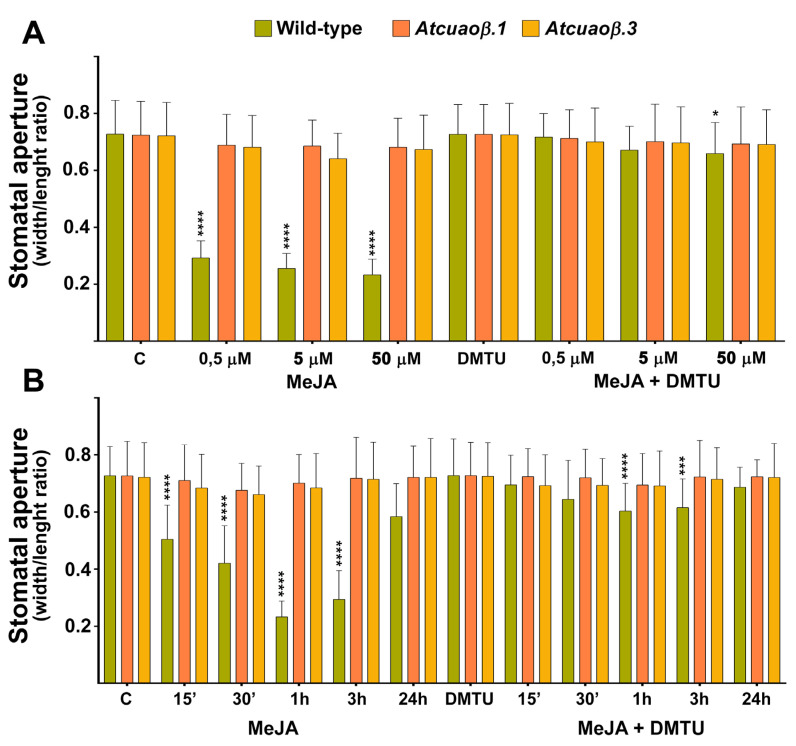
Effect of three different concentrations of MeJA and *N,N*′-dimethylthiourea (DMTU) (**A**) and time course analysis of the effect of MeJA and DMTU (**B**) on stomatal pore width/length ratio of seven-day-old WT, *Atcuaoβ.1* and *Atcuaoβ.3* mutant seedlings. Seedlings were treated for 1 h with 0.5, 5, or 50 μM MeJA and 100 μM DMTU, either alone or in combination with each hormone concentration (**A**). Seedlings were treated for 15, 30 min, 1, 3 and 24 h with 50 μM MeJA and 100 μM DMTU, either alone or in combination with the hormone (**B**). Mean values ± SD (*n* = 15) are reported. The significance levels between WT control plants and WT treated plants are reported. *p* levels have been calculated with one-way ANOVA analysis; *p* levels > 0.05; *, ***, and **** *p* levels are equal to or less than 0.05, 0.001 and 0.0001, respectively.

WT, *Atcuaoβ.1* and *Atcuaoβ.3* seedlings were treated for 1 h with 0.5, 5 or 50 μM MeJA to evaluate the effect of three different MeJA concentrations on the modulation of stomatal aperture by measuring the width/length ratio of the stomatal pore (Figure 4A). In control conditions, no significant differences in stomatal aperture levels were found between seven-day-old WT and *Atcuaoβ* mutants. The dose–response curve (Figure 4A) relative to the effects of MeJA supply on the modulation of stomatal aperture revealed 50 μM MeJA as optimal concentration. In detail, dose–response curve analysis shows that stomata guard cells of WT 0.5, 5 and 50 μM MeJA-treated plants displayed a stomatal pore closure of 60%, 65% and 70%, respectively, in respect to untreated plants after 1 h (Figure 4A). Instead, both *Atcuaoβ* loss-of-function mutants did not show any differences in stomatal aperture levels upon all concentrations of MeJA-treatment, displaying the same value of width/length ratio in comparison to both WT and mutant untreated plants. Furthermore, the H_2_O_2_ scavenger *N-N^1^*-dimethylthiourea (DMTU) at the concentration of 100 μM reversed completely the 0.5 and 5 μM MeJA-induced stomatal closure in WT plants, restoring the pore width/length ratio at the level of the untreated plants, while reversed almost completely (90%) the 50 μM MeJA-induced stomatal closure in WT plants, in respect to untreated plants. In addition, DMTU treatment did not significantly affect stomatal aperture under control conditions in WT plants or in MeJA-treated and -untreated mutants (Figure 4A).

Concerning the time course analysis (Figure 4B), experiments were focused on the effect of 50 μM MeJA on the modulation of stomatal aperture by measuring the width/length ratio of the stomatal pore of WT, *Atcuaoβ.1* and *Atcuaoβ.3* seedlings treated for 15, 30 min, 1, 3 and 24 h (Figure 4B). In control conditions, no significant differences in stomatal aperture levels were found between seven-day-old WT and *Atcuaoβ* mutants. Time course analysis shows that stomata guard cells of WT MeJA-treated plants started to close after 15 min and displayed a closure peak of 65% in respect to untreated plants after 1 h (Figure 4B). Instead, both *Atcuaoβ* loss-of-function mutants did not show any differences in stomatal aperture levels upon MeJA-treatment, displaying the same value of width/length ratio of stomatal pore in comparison to both WT and mutant untreated plants. Moreover, 100 μM DMTU treatment reversed almost completely the MeJA-induced stomatal closure in WT plants restoring the pore aperture at about 90% in respect to untreated plants after 1–3 h of treatment, whereas it did not significantly affect stomatal aperture under control conditions in WT plants or in MeJA-treated and -untreated mutants (Figure 4B).

**Figure 5:**
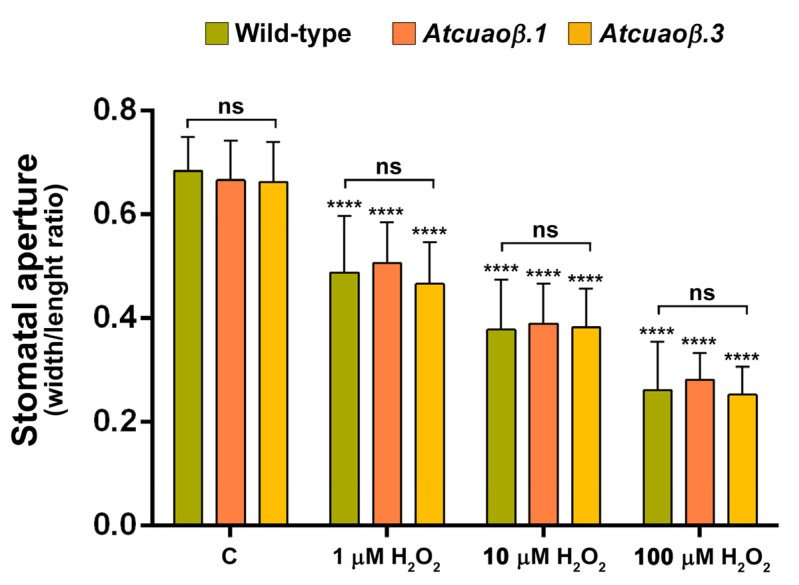
Effect of three different concentrations of H_2_O_2_ on stomatal pore width/length ratio of seven-day-old WT, *Atcuaoβ.1* and *Atcuaoβ.3* mutant seedlings. Seedlings were treated for 1 h with 1, 10, or 100 μM H_2_O_2_. Mean values ± SD (*n* = 15) are reported. The significance levels between the treated plants and the corresponding untreated plants are reported for each treatment, while the significance levels between the WT and *Atcuaoβ* mutants supplied with the same concentration of H_2_O_2_ (horizontal square brackets) are reported. *p* levels have been calculated with one-way ANOVA analysis; *p* levels > 0.05; ****, *p ≤* 0.0001; ns: non-significant.

Considering the H_2_O_2_ key role suggested by DMTU-reversion of MeJA-induced *AtCuAOβ*-mediated stomatal closure, the effect of exogenous H_2_O_2_ on stomatal aperture was analysed by treating seven-day-old WT, *Atcuaoβ.1* and *Atcuaoβ.3* seedlings for 1 h with 1, 10 or 100 μM H_2_O_2_. As displayed in Figure 5, exogenous H_2_O_2_ was effective in inducing stomatal closure in a dose-dependent manner, at the same extent on WT, *Atcuaoβ.1* and *Atcuaoβ.3* seedlings, for each tested concentration. In particular, stomata of both WT and *Atcuaoβ* mutants displayed same closure levels of about 30%, 50% and 60% upon treatment, respectively with 1, 10 and 100 μM H_2_O_2_, as compared to untreated plants, demonstrating the exclusive H_2_O_2_ role in signalling downstream of the MeJA-induced CuAO-mediated PA oxidation.

**Figure 6:**
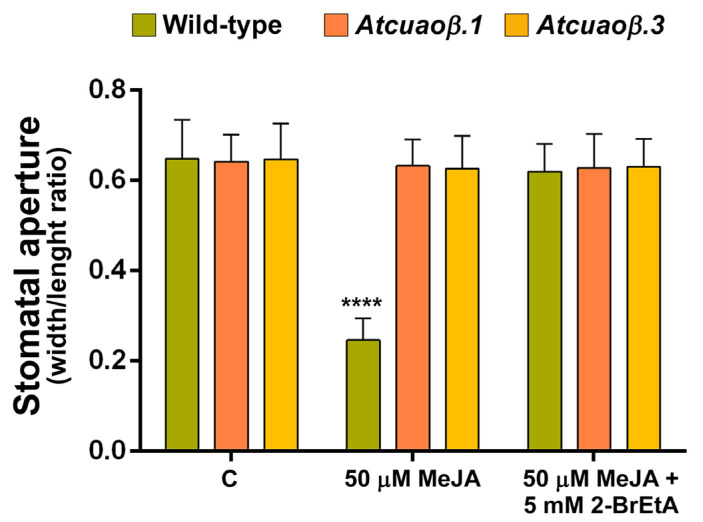
Effect of MeJA and CuAO inhibitor 2-BrEtA on stomatal pore width/length ratio of seven-day-old WT, *Atcuaoβ.1* and *Atcuaoβ.3* mutant seedlings. Seedlings were treated for 1 h with 50 μM MeJA alone or in combination with 5 mM 2-BrEtA. Mean values ± SD (*n* = 15) are reported. The significance levels between WT control plants and WT treated plants are reported. *p* levels have been calculated with one-way ANOVA analysis; *p* levels > 0.05; ****, *p ≤* 0.0001.

Consistently with these data, treatment with the CuAO-specific inhibitor 2-bromoethylamine (2-BrEtA) blocked the MeJA-mediated stomatal closure in WT plants, restoring the stomatal pore aperture at the same values of the untreated plants, whereas it did not significantly affect stomatal aperture in MeJA-treated *Atcuaoβ* mutants (Figure 6).

### 3.4. Stomatal Closure Induced by MeJA Is Associated with a ROS Level Increase in Guard Cells Mediated by AtCuAOβ

**Figure 7:**
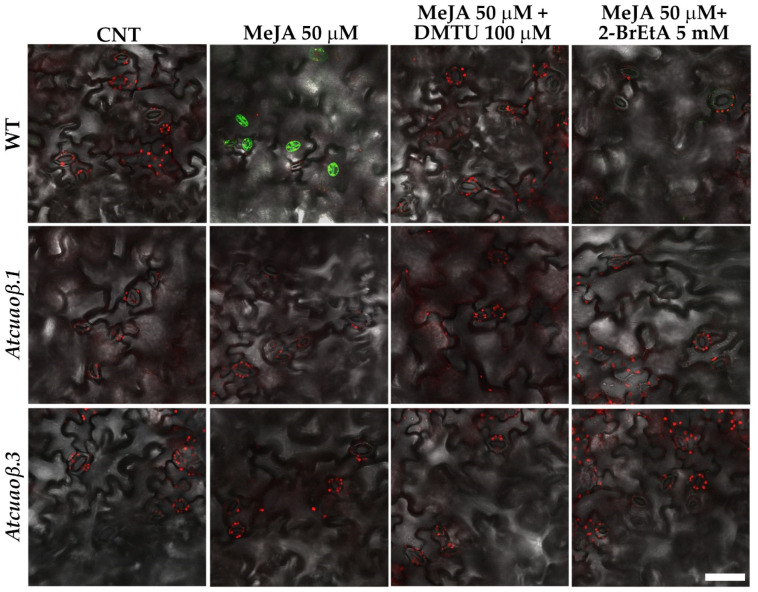
ROS levels in guard cells of cotyledonary leaves from seven-day-old seedling. In situ, ROS detection by LSCM analysis after CM-H_2_DCFDA staining of cotyledonary leaves from WT, *Atcuaoβ.1* and *Atcuaoβ.3* mutant seedlings, untreated or treated with 50 µM MeJA, either alone or in combination with 100 µM DMTU or 5 mM 2-BrEtA. Micrographs are representative of those obtained from five independent experiments, each time analysing cotyledonary leaves from five seedlings per genotype and treatment. Bar = 50 µm.

To further investigate the contribution of AtCuAOβ in the mediation of stomatal closure through the MeJA-induced H_2_O_2_ production, ROS levels in guard cells were visualized by Laser Scanning Confocal Microscopy (LSCM) using a chloromethyl derivative of 2′,7′-dichlorodihydrofluorescein diacetate (CM-H_2_DCFDA). WT and *Atcuaoβ* mutant seedlings were treated with 50 µM MeJA either alone or in combination with 100 µM DMTU or 5 mM 2-BrEtA. 50 µM MeJA-treatment induced an increase in ROS levels in guard cells of WT plants, as indicated by an intense CM-H_2_DCFDA fluorescence, that was reversed by DMTU or 2-BrEtA treatment, while ROS were undetectable in untreated WT and both untreated and treated mutant seedlings (Figure 7).

## 4. Discussion

Plant-environment interaction, which includes responses to stress conditions such as drought, wounding, heat, cold as well as pathogen infection, has been the subject of in-depth studies due to its huge economic and agricultural implications. These stress responses are dependent on integrated transduction pathways involving different signalling molecules such as ROS, Ca^2+^, NO, phytohormones and other signalling components, that orchestrate plant responses to biotic and abiotic stress modulating metabolism, proteomic and transcriptomic variations to allow plant acclimation and survival [20]. The integration of different signalling pathways that leads to plant acclimation should be finely coordinated in order to respond adequately to the multitude and to the different intensity of the stress types. In this context, one of the most important defence mechanisms induced by both biotic and abiotic stress is the modulation of the stomatal aperture.

Stomatal pores are microscopic gates in plant epidermis formed between two guard cells that create a passage for the exchange of carbon dioxide (CO_2_) and water vapour (H_2_O) between plants and the atmosphere and have long been recognized as a major point of entry for plant pathogenic bacteria [21]. Historically, these surface openings were considered as passive gateways, however several studies have shown that stomata can play an active role in limiting bacterial invasion and prevent water loss under stress conditions [22,23]. It is well known that biotic/abiotic stresses such as herbivory attack, pathogen infection, drought, and wounding trigger stomatal closure in different plant species [24,25,26,27]. Indeed, stomatal closure has been reported to be involved in limiting water loss under herbivory [28]. Chewing and sucking herbivores cause open wounds that compromise the vascular tissues and may interfere with transpiration. These events can lead to the accumulation of the drought-associated phytohormone ABA and of the wound-associated phytohormone MeJA, triggering stomatal closure [28]. ABA and MeJA accumulate in guard cells causing the production of NO and H_2_O_2_, the subsequent increase of intracellular Ca^2+^ concentration, which in turn controls the activity of ion channels and leads to a decrease in the osmotic pressure, which results in H_2_O efflux and finally in stomatal closure [1]. In this context, H_2_O_2_ delivered from AO-mediated PA catabolism could play a role as an important mediator in stress-induced stomatal closure.

The Arabidopsis *CuAOβ* encodes an apoplastic CuAO expressed in leaf guard cells and root protoxylem tissues [8,9,10]. Here, its role in stomatal closure signalled by MeJA was explored by pharmacological and genetic approaches. Data herein reported confirm a role for AtCuAOβ in MeJA-induced stomatal closure. Coherently, data show a strong *AtCuAOβ* expression in guard cells of different tissues/organs in different developmental stages (Figure 1) thus suggesting that this gene could play a pivotal role in the modulation of stomatal pore aperture. Moreover, the induction of its expression levels by the wound-associated hormone MeJA (Figure 2), especially in stomata guard cells (Figure 3), suggests a possible role in wound-induced MeJA-mediated stomatal closure, further supported by both the unresponsiveness of *Atcuaoβ* loss-of-function mutants (Figure 4) and the 2-BrEtA-reversion (Figure 6) of MeJA-induced AtCuAOβ-mediated stomatal closure in WT plants.

Results herein reported also indicate the involvement of H_2_O_2_ delivered by AtCuAOβ-mediated PA catabolism in MeJA-induced stomatal closure. In this regard, treatment with the H_2_O_2_ scavenger DMTU 100 μM reversed completely the 0.5 and 5 μM MeJA-induced stomatal closure in WT plants, while reversed almost completely the 50 μM MeJA-induced stomatal closure in WT plants, restoring the pore aperture at the 90% in respect to untreated plants. A possible hypothesis that explains this partial reversion of the stomatal closure in WT plants could be the ineffectiveness of the DMTU in completely removing the H_2_O_2_ produced after treatment with a higher concentration of MeJA. Moreover, a still different hypothesis that explains the partial reversion could be the co-occurrence of H_2_O_2_-independent mechanisms involving other components directly connected with CuAO activity, i.e., the aminoaldehyde production or changes in the PA levels. However, the exclusive H_2_O_2_ role in signalling downstream of the MeJA-induced CuAO-mediated PA oxidation, was strongly supported by H_2_O_2_ effectiveness in inducing stomatal closure at the same extent in both WT and *Atcuaoβ* mutants. As shown in Figure 5, 1 h treatment with 100 μM H_2_O_2_ induced 60% stomatal closure in all three genotypes, which is comparable to the effect of 0.5 μM MeJA on WT plants. If MeJA-induced CuAO-mediated PA oxidation was even partly signalled by H_2_O_2_-independent mechanisms, the extent of stomatal closure in WT and *Atcuaoβ* mutants would be different and, under these circumstances, mutant plants would be lacking not only H_2_O_2_ but also some other necessary pathway ensuring stomatal closure.

Coherently, MeJA prompted H_2_O_2_ production during stomatal closure, as visualized by LSCM after CM-H_2_DCFDA staining, reversible by both DMTU and 2-BrEtA and not detectable in guard cells of open stomata of MeJA-treated *Atcuaoβ* mutants.

Among AtCuAO family members, in addition to the apoplastic AtCuAOβ herein reported, the vacuolar AtCuAOδ and the peroxisomal AtCuAOζ have been shown to be expressed in guard cells and involved in the control of ABA-induced stomatal movement regulation [3,10,29]. Moreover, in Arabidopsis other several components have been identified as ROS sources involved in the complex signal transduction pathway which leads to the stomatal movement regulation under stress condition, among which there are the plasma membrane resident NADPH oxidases [30] and the OST1 protein kinase [31]. Furthermore, it has been shown that peroxisomal AtPAO cross-talks with NADPH oxidase to activate mitochondrial alternative oxidase, highlighting the complexity of ROS biosynthesis and homeostasis [32]. The identification of multiple pathways involving different enzymatic systems and subcellular compartments required for the stomatal movements control in Arabidopsis suggests that this complex mechanism is strongly coordinated by a not completely clear network, in which a stress-specific hormonal control is needed to induce the activation of ROS sources with different stress-specific sensitivity.

Understanding these mechanisms represents a key step in order to set up potential application approaches to feature agricultural crops in the actual environmental context, in which adverse climatic conditions are becoming increasingly relevant.

## Figures and Tables

**Table 1:** Primers used for identification of the T-DNA insertional loss-of-function *Atcuaoβ.3* mutants by PCR analysis.

Name of Primer	Sequence of Primer
*LBa1*	5′-GATGGTTCACGTAGTGGGCCATCGC-3′
*RP-Atcuaoβ.3*	5′-ATCACTATAAAACCCACCGGC-3′
*LP-Atcuaoβ.3*	5′-ACGTTCATGGACATTGGAGAG-3′

**Table 2:** Primers used for RT-qPCR analysis on whole Arabidopsis seedlings after MeJA treatments.

Name of Primer	Sequence of Primer
*UBC21-for*	5′-CTGCGACTCAGGGAATCTTCTAA-3′
*UBC21-rev*	5′-TTGTGCCATTGAATTGAACCC-3′
*AtCuAOβ-qPCR-for*	5′-CAAGTGGGGAAGCTGAAATAAGTTTAGTG-3′
*AtCuAOβ-qPCR-rev*	5′-TCCTCCGAGAAGACGTTTTGTTAACTTC-3′

## Data Availability

Not applicable.

## Supplementary Materials

The following are available online at https://www.mdpi.com/article/10.3390/cells10123399/s1, Figure S1. Characterization of the T-DNA insertional mutant for *AtCuAOβ* (TAIR accession number: 2129519), Figure S2. Validation of treatment in liquid medium method to confirm the stable maintenance in different solutions (liquid medium, LM; liquid medium without sucrose, LM^-suc^; distilled water, H_2_O) of the stomatal aperture induced by the opening solution (OS), Figure S3. Control condition of the *AtCuAOβ* tissue-specific expression pattern analysis upon treatment with 50 μM MeJA showed in Figure 3.
