# Supplementary material for: A New Player in Jasmonate-Mediated Stomatal Closure: The Arabidopsis thaliana Copper Amine Oxidase β
Source: Cells. 2021 Dec 2;10(12):3399. doi: 10.3390/cells10123399 (PMC8699484; doi:10.3390/cells10123399)
Supplement: Supplementary file 1 [file cells-10-03399-s001.zip › cells-1426579-SI.pdf]

Supplementary Materials

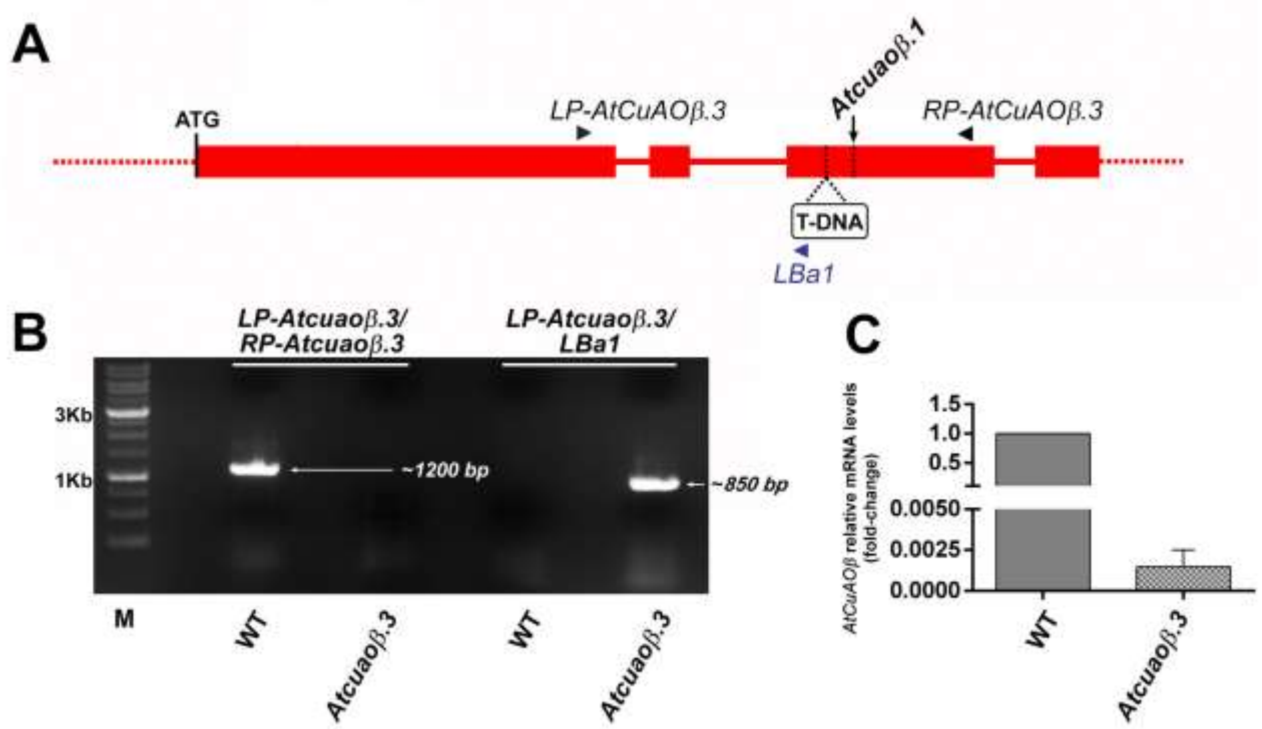

Figure S1. Characterization of the T-DNA insertional mutant for *AtCuAOβ* (TAIR accession number: 2129519). Schematic representation of the T-DNA insertion site in the *Atcuaoβ.3* (SALK_082394.32.30.X line TAIR accession number: 1005822711) mutant (**A**). *LP-Atcuaoβ.3*/*RP-Atcuaoβ.3*: gene specific primers; *LBa1*: T-DNA left border specific primer for SALK T-DNA insertion lines. Genotyping of *Atcuaoβ.3* mutant (**B**). In homozygous *Atcuaoβ.3* seedlings, T-DNA insertion in both alleles was demonstrated by the presence of the specific PCR fragment with *LP-AtCuAOβ.3*/*LBa1* primers and the absence of amplification with *LP-Atcuaoβ.3*/*RP-Atcuaoβ.3* primers. Total DNA from WT was used as control. M: DNA marker. RT-qPCR analysis of total RNA from WT and *Atcuaoβ.3* mutant seedlings (**C**). Gene expression was analyzed in 7-day-old WT and *Atcuaoβ.3* mutant seedlings. The reported values of *AtCuAOβ* expression fold-change of *Atcuaoβ.3* mutant seedlings are relative to the corresponding expression values of WT plants, which were assumed to be one. Data are the result of three biological replicates, each with three technical replicates (mean values ± SD; $n = 3$).

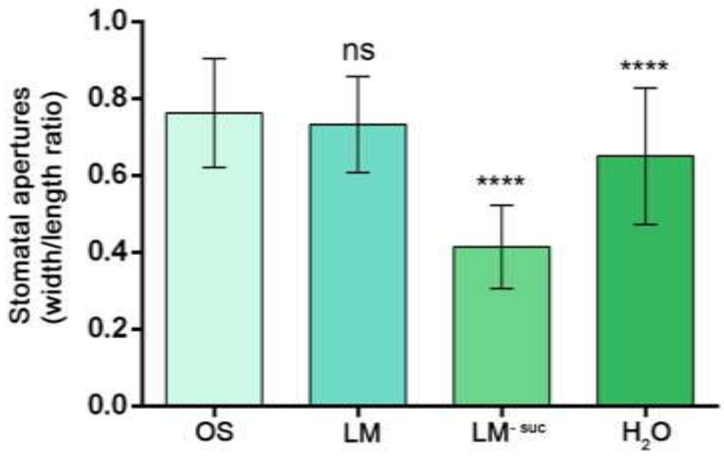

Figure S2. Validation of treatment in liquid medium method to confirm the stable maintenance in different solutions (liquid medium, LM; liquid medium without sucrose, LM^{-suc}; distilled water, H_2O) of the stomatal aperture induced by the opening solution (OS). Unlike Jung *et al*, treatments were done in fresh LM instead of directly in the OS and the variation of the method was validated with a series of pilot experiments to confirm the stable maintenance of the stomatal aperture levels in different solutions. 7-day-old WT seedlings grown on solid medium were incubated in OS for 3 h under light to allow stomatal opening. Then, seedlings were incubated for 24 h under light in fresh OS, LM, LM^{-suc} and H_2O to analyze the differences of stomatal aperture variation (width/length ratio). Mean values ± SD (n = 15) are reported. The significance levels between control plants in OS and plants in the other solutions are reported. P levels have been calculated with one-way ANOVA analysis; P levels > 0.05; **** P levels are equal to or less than 0.0001; ns, not significant.

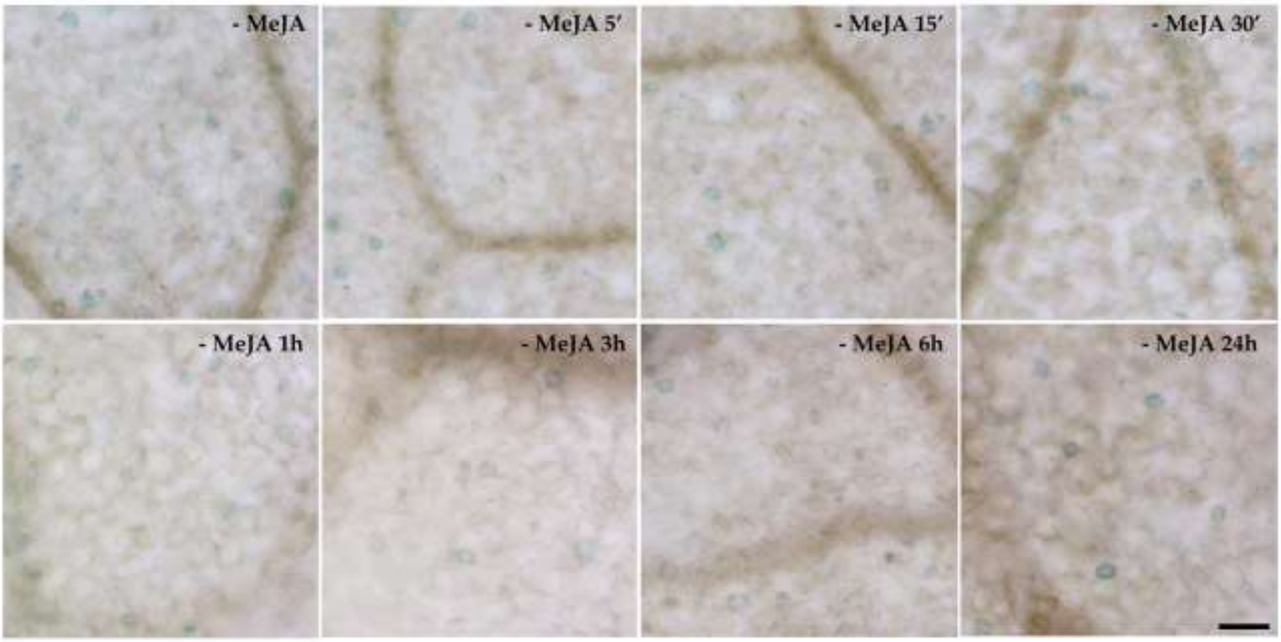

Figure S3. Control condition of the *AtCuAOβ* tissue-specific expression pattern analysis upon treatment with 50 μM MeJA showed in Figure 3. Light microscopy analysis by GUS staining in cotyledons of 7-day-old *AtCuAOβ::GFP-GUS* transgenic seedlings untreated (Figure S3) or treated (Figure 3) for 5 min, 15 min, 30 min, 1 h, 3 h, 6 h and 24 h. The staining reaction proceeded for 2 h. Micrographs are representative of those obtained from 15 leaves from 3 independent experiments. Bar = 50 μm.
